# Evaluation of the expression and clinical value of lncRNA AC010761.9 in human gastric adenocarcinoma

**DOI:** 10.1186/s12957-017-1289-y

**Published:** 2018-03-02

**Authors:** Zhihua Wang, Kai Wang, Yuan Dang, Xiaojuan Ouyang, Fan Zhang, Wenyuan Wang, Lie Wang, Qiaojia Huang

**Affiliations:** 1 Department of Experimental Medicine, Fuzong Clinical Medical College, Fujian Medical University, Fuzhou, China; 2 Department of Experimental Medicine, Fuzhou General Hospital, 156 North Xi-er Huan Road, Fuzhou, Fujian 350025 China; 3 Department of General Surgery, Fuzong Clinical Medical College, Fujian Medical University, Fuzhou, China; 4 Department of General Surgery, Fuzhou General Hospital, Fuzhou, Fujian 350025 China; 5 Department of Clinical Laboratory of the 92th Hospital of PLA, North Binjiang Road, Nanping City, Fujian 353000 China

**Keywords:** Gastric adenocarcinoma, lncRNA AC010761.9, Expression and clinical value

## Abstract

**Background:**

The current study determined the expression and clinical value of lncRNA AC010761.9 in human gastric adenocarcinoma (GA).

**Methods:**

Real-time quantitative reverse transcription (qRT)-PCR was used to detect the level of lncRNA expression in 145 GA tissues and three GA cell lines, and the correlation between its level and clinicopathologic characteristics and potential corresponding mRNA of TNF receptor-associated factor 4 gene (*TRAF4*) was then evaluated.

**Results:**

Elevated lncRNA AC010761.9 was detected in all 6 GA tissues by previous lncRNA expression profile microarray assay. LncRNA AC010761.9 was over-expressed in 99 of 145 GA tissues (68.3%) with an elevated fold change of up to 35.14 compared to matched paracancerous tissues (*p* < 0.05), and was also over-expressed in the 3 GA cell lines (MGC803, BGC823, and SGC7901) compared to the normal gastric mucosal epithelial cell line (GES-1 cells; *p* < 0.05) by qRT-PCR. The elevated expression of this lncRNA was related to tumor size (*p* = 0.028), degree of differentiation (*p* = 0.047), and serum carbohydrate antigen (CA19-9) and carcinoembryonic antigen (CEA) concentrations (*p* = 0.026 and *p* = 0.037, respectively). Multivariate analysis further confirmed that the expression of lncRNA AC010761.9 was related to the degree of tumor differentiation (*p* = 0.015). Additionally, the expression of lncRNA AC010761.9 had a positive correlation with the mRNA expression of the potentially associated gene (TRAF4) in GA tissues (*r* = 0.385, *p* < 0.01).

**Conclusions:**

LncRNA AC010761.9 may be linked to GA progression and is a potential new biomarker for GA.

**Electronic supplementary material:**

The online version of this article (10.1186/s12957-017-1289-y) contains supplementary material, which is available to authorized users.

## Background

Globally, gastric cancer is one of the most common malignant tumors affecting the human digestive tract [1]. Gastric cancer is also one of the major leading causes of cancer-related deaths worldwide [1]. Moreover, patients with advanced-stage gastric cancer frequently die. This malignant illness can occur at any age; however, gastric cancer usually affects the elderly. Data from cancer epidemiologic investigations have shown that the incidence of gastric cancer has a trend towards younger patients in recent years, thus it is important to find more effective and available biomarkers to further understand the pathogenesis of gastric cancer and to guide therapeutic decisions. Cancer biomarkers are closely related to the features present in malignant cells, which often exhibit great value in reflecting the occurrence and development of the tumor or monitoring the tumor response to treatment [2]. If biomarkers for DNA, RNA, and protein can be used clinically, the features of the malignancies from multiple levels will be demonstrated and may be of importance in guiding therapy. An evaluation of potential new biomarkers will help reach the above mentioned goals.

Recent studies have shown that long non-coding RNAs (lncRNAs) are closely related to a variety of human cancer occurrence and development [3–5]. LncRNAs are RNAs without a function on coding proteins and are named based on length (i.e., > 200 nucleotides) [6]. The roles for lncRNAs in cancer have attracted attention in the past several years, and many studies have reported that lncRNAs are a new class of biomarkers for human cancers, including gastric cancer [3, 7]. Emerging evidence has shown that gastric cancer occurrence and progression are accompanied by alterations in lncRNA expression or function [8, 9], so lncRNAs have been shown to be able to serve as biomarkers for the diagnosis and/or therapy of gastric cancer [10]; however, the actual number of lncRNAs related to gastric cancer is unknown.

LncRNA AC010761.9 is a natural antisense lncRNA which has a length of 521 nucleotides and does not have a function to translate protein. This lncRNA is located on human chromosome 17, and the potentially associated gene of this lncRNA is thought to be TNF receptor-associated factor (*TRAF*) 4, a gene which is able to promote cancer development via different molecular mechanisms [11, 12]. According to the results from our previous lncRNA expression profile chip assay, lncRNA AC010761.9 was identified to be up-regulated in all six gastric adenocarcinoma (GA) tissues compared to the levels in the matched paracancerous tissues. No studies have been conducted on this lncRNA with gastric cancer previously, so this lncRNA was selected for further investigation in this work. GA is a primary malignant tumor of the stomach. Therefore, in the present study, lncRNA AC010761.9 expression in 145 GA tissues and the matched paracancerous tissues and in three different GA cell lines (BGC-823, SGC-7901, and MGC-803 cells) was determined, and the clinical value was evaluated according to the levels of expression and clinicopathologic factors. In addition, the relationship between lncRNA AC010761.9 and TRAF4 mRNA expression in 78 samples and three GA cell lines was then investigated.

## Methods

### Collection of GA tissue specimens

One hundred forty-five fresh GA tissues were collected at Fuzhou General Hospital in Fujian, China, between March 2014 and October 2015. This group of patients included 145 patients, all of whom had a definite diagnosis of primary GA. The tissue samples, including GA and the matched paracancerous tissues (5 cm from the lesion), were obtained immediately after the surgical procedure, which was performed to remove the primary GA, kept in RNAlater (Qiagen, Duesseldorf, Germany), and stored at − 80 °C before use. The histopathologic diagnosis, the staging tumor-node-metastasis (TNM), and the determination of the histologic grade were performed by two pathologists working in the Department of Pathology of Fuzhou General Hospital (Fujian, China) according to the International Union Against Cancer (5th edition) and the guidelines of the National Comprehensive Cancer Network (NCCN) Clinical Practice of Oncology (V.1.2011) [13]. No pre-operative chemotherapy or radiotherapy was administered. The clinical and pathologic data of this group of patients were also collected and were shown in Table 1.

**Table 1:** Relationships between lncRNA AC010761.9 expression (ΔCt value) and patient clinical pathologic factors and serum tumor markers

Characteristics	No. of case (%)	Mean ± SD	*p* value
Age			
≥ 60	70 (54.2)	19.08 ± 2.904	0.162
< 60	59 (45.8)	18.31 ± 3.309	
Gender			
Male	102 (79.1)	18.66 ± 3.087	0.620
Female	27 (20.9)	18.99 ± 3.227	
Tumor location			
Upper	35 (28.0)	19.33 ± 3.198	0.621
Middle	48 (38.4)	18.52 ± 3.062	
Lower	42 (33.6)	18.68 ± 3.140	
Diameter			
≥ 4	70 (54.2)	19.28 ± 2.967	*0.028*
< 4	59 (45.8)	18.07 ± 3.167	
Differentiation			
Poor	97 (75.2)	18.48 ± 3.096	*0.047*
Moderate	30 (23.2)	19.73 ± 2.966	
Well	2 (1.6)	15.39 ± 1.478	
Invasion			
T1	11 (8.5)	18.08 ± 3.155	0.269
T2	11 (8.5)	18.76 ± 4.302	
T3	14 (10.8)	17.36 ± 3.074	
T4	93 (72.2)	19.01 ± 2.931	
Lymphatic metastasis			
N0	32 (24.8)	18.98 ± 3.274	0.597
N1–3	97 (75.2)	18.64 ± 3.063	
Venous invasion			
Absent	71 (55)	18.67 ± 3.184	0.808
Present	58 (45)	18.80 ± 3.037	
Perineural invasion			
Absent	59 (45.7)	18.51 ± 3.243	0.399
Present	70 (54.3)	18.98 ± 2.946	
CEA			
Normal	109 (84.5)	18.48 ± 2.935	*0.037*
High	20 (15.5)	20.05 ± 3.727	
CA19-9			
Normal	113 (87.5)	18.50 ± 2.999	*0.026*
High	16 (12.5)	20.34 ± 3.469	
AFP			
Normal	121 (93.8)	18.64 ± 3.082	0.248
High	8 (6.2)	19.96 ± 3.444	

The italicized values are significant at *p* < 0.05

(Except patients in the subgroup related with tumor location only had 125 cases with detailed clinical information, other subgroups had 129 patients with detailed clinical information).

### Preparation of GA tissue specimens for the immunohistochemical assay

The immunohistochemical (IHC) assay used paraffin-embedded tissue samples, which were made using the same fresh tissue samples described above and performed by two pathologists.

### Collection of serum samples

The serum samples from the same patients were collected by laboratory technicians prior to surgery and used for measurement of the levels of digestive tract malignant tumor markers (AFP, CEA, and CA19-9).

### LncRNA expression profile microarray chip assay

The results from the Human LncRNA Expression Profile Microarray V3.0 (Arraystar, Rockville, MD, USA) were obtained from the previous work and completed by Kang Chen Bio-tech (Shanghai, China) based on the manufacturer’s instructions [13]. Microarray assay and data analysis were performed, from which the differentially expressed lncRNAs and mRNAs from six GA tissues and the matched paracancerous tissues were identified [13], by KangChen Bio-tech [13].

### Culture of GA and normal gastric epithelial cells

The three GA cell lines used in the current study were BGC-823, SGC-7901, and MGC-803. BGC-823 and MGC-803 were obtained from the Shanghai Institute of Biochemistry of the Chinese Academy of Sciences in Shanghai, China, and SGC-7901 was obtained from the American Type Culture Collection (Manassas, VA, USA). The normal cell line used in this work was the GES-1 cell line, which was a normal human gastric mucosal epithelial cell line, and was purchased from the Beijing Cancer Institute (Beijing, China). The cells were cultured in RPMI-1640 medium (for BGC-823, SGC-7901, and MGC-803 cells) and Dulbecco’s modified Eagle’s medium (for GES-1 cells; Invitrogen, Grand Island, NY, USA) based on our previously reported methods [13].

### Isolation of total RNA and performance of qRT-PCR

Both the total RNA from the tissues and cultured cells were extracted with the TransZol Up Plus RNA Kit (Transgen Biotech Company, Beijing, China) according to the instructions provided by the manufacturer. Reverse transcription of mRNA into cDNA was then performed with a GoScript™ Reverse Transcription System (Promega, Madison, WI, USA) according to the instructions provided by the manufacturer. Amplification of the products with the SYBR Green Mix kit (GoTaq® qPCR Master Mix; Promega) was carried out in a 2720 Thermal Cycler PCR System (ABI, Grand Island, NY, USA). The primers used in the amplification included that for internal reference 18s RNA, lncRNA AC010761.9, and TRAF4 mRNA, which were as follows: forward 5′-GTAACCCGTTGAACCCCATT-3′ and reverse 5′-CCATCCAATCGGTAGTAGCG-3′ (for 18s RNA); forward 5′-GAGGGAACACTTCTTGCGGG-3′ and reverse 5′-CAGGGCCAGTGTCAACCAAA-3′ (for lncRNA AC010761.9); and forward 5′-CCACCGTTTCTGCGATACCT-3′ and reverse 5′-GGGTCTGGGTAGATCTTGGC-3′ for TRAF4 mRNA. The PCR amplification conditions contained the following three steps: step 1, 95.0 °C for 2 min; step 2, 95.0 °C for 15 s and 58 °C for 40 s (total 40 cycles); and step 3 (dissociation stage), 95 °C for 15 s, 60 °C for 15 s, and 95 °C for 15 s. The cycle threshold (Ct) values of 18s RNA (control), lncRNA AC010761.9, and TRAF4 mRNA were automatically recorded by the machine. The level of lncRNA AC010761.9 and TRAF4 mRNA expression was obtained by calculating the ΔCt, which were inversely proportional to the level of expression. Samples with a higher ΔCt meant lower AC010761.9 or TRAF4 expression. The relative fold changes of AC010761.9 or TRAF4 expression in GA tissues versus matched non-GA tissues or in GA cell lines versus normal gastric cell lines (GES-1) were obtained by calculating the formula (2−^^Ct). The results were obtained from three independent experiments and expressed as the means ± SD.

### Quantitative examination of serum AFP, CEA, and CA19-9 levels

Quantitative examinations of serum carcinoembryonic antigen (CEA), carbohydrate antigen 19–9 (CA19-9), and alpha-fetoprotein (AFP) concentrations in 129 patients with GA were part of the routine work-up for GA patients, which were performed by technicians before surgery with the Quantitative Kit for Tumor Marker (Protein Chip-Chemiluminescence; HealthDigit, Huzhou, China) with the HD-2001A ChipReader System (HealthDigit) [13]. Individuals with < 5.0 ng/ml for CEA, < 35.0 ng/ml for CA19-9, and < 20.0 ng/ml for AFP were regarded as having low (normal) expression of the three digestive tract tumor markers.

### Immunohistochemical assay

There were ten immunohistochemical markers to be routinely performed for patients with GA in this hospital (vascular endothelial growth factor [VEGF], human epidermal growth factor receptor 2 [C-erbB-2 or HER2], thymidylate synthase [TS], breast cancer 1 [BRCA1], excision repair cross-complementation group 1 [ERCC1], Ki67 antigen [Ki67], ribonucleotide reductase subunit M1 [RRM1], synaptophysin [Syn], neuronal cell adhesion molecule 1 [CD56], and chromogranin A [CgA]). The data were collected from the medical records. The methods, reagents used for IHC assays, and the result determination were the same as previously described [13]. The performances of the IHC assay and result analysis were conducted by pathologists working in this hospital.

### Statistical analysis

SPSS 17 was used for statistical analysis. Data are expressed in the form of mean ± standard deviation. The comparison between the groups was performed using a *t* test and one-way ANOVA analysis. The relationship between the levels of lncRNA AC010761.9 and TRAF4 mRNA expression was analyzed using Pearson correlation analysis. Multivariate logistic regression analysis was further used to assess the relationships between lncRNA AC010761.9 expression and patient clinical pathologic factors, and factors used in the multivariate analysis included age, gender, tumor location, diameter, differentiation grade, invasion, lymphatic metastasis, venous invasion, and perineural invasion. Multivariate models did not adjust for that factor which was being tested. A *p* < 0.05 was taken as statistically significant.

## Results

### LncRNA expression chips showed that lncRNA AC010761.9 is over-expressed in GA tissues

**Fig. 1:**
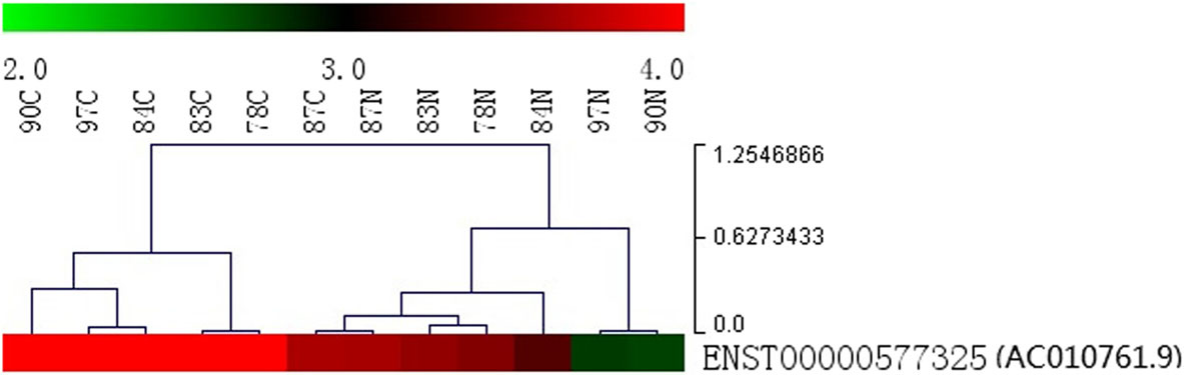
LncRNA AC010761.9 was over-expressed in GA tissues by lncRNA expression chip assay. *T* cancer tissues, *N* matched non-cancer tissues. Cluster analyses from the six GA and their paired non-GA tissues lncRNA chip results showed that LncRNA AC010761.9 was over-expressed in GA tissues compared with that in the paired non-GA tissues (mean increased fold = 2.01 times, *p* < 0.05)

The lncRNA microarray chip assays were performed in six GA tissues and six matched paracancerous tissues. The results showed that the level of lncRNA AC010761.9 expression was higher in six GA tissues than the matched non-GA tissues (mean increased fold was 2.01, *p* < 0.05) (Fig. 1). LncRNA AC010761.9 may be a dysregulated lncRNA in GA, thus it was considered to warrant further investigation.

### qRT-PCR confirmed that lncRNA AC010761.9 is over-expressed in GA tissues and cell lines

**Fig. 2:**
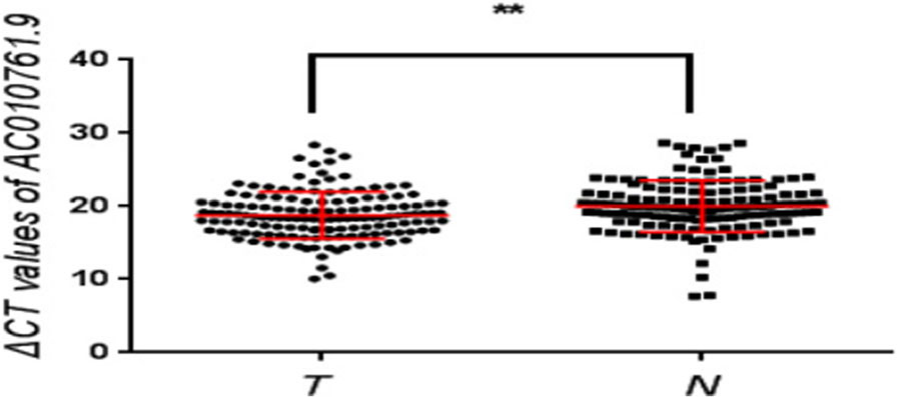
LncRNA AC010761.9 was over-expressed in GA tissues by quantified RT-PCR measurement. *T* cancer tissues, *N* matched non-cancer tissues. T versus N, *p* < 0.01. The data were from 145 cases of GA. The higher the ΔCt values, the lower the lncRNA AC010761.9 expression. Data were obtained from three independent tests

**Fig. 3:**
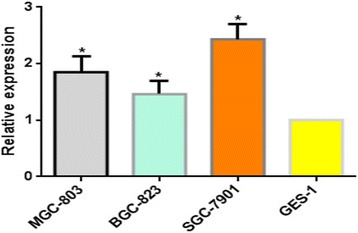
LncRNA AC010761.9 was over-expressed in GA cell lines. The data were from three GA cell lines (MGC-803, BGC-823, and SGC-7901) and control cells (normal gastric cell line [GES-1]). GA cells versus control cells (all *p* < 0.05). The higher the ΔCt values, the lower the lncRNA AC010761.9 expression. Data were obtained from three independent tests

As depicted in Fig. 2, the results of qRT-PCR further showed that the level of lncRNA AC010761.9 expression was higher in 99 of 145 GA tissues than the matched non-GA tissues, with an over-expressed rate of 68.3% (*p* < 0.01). When the mean level of expression of this lncRNA in the matched non-GA tissues was taken as 1, an elevated fold up to 35.14 in GA tissues was observed. The results of qRT-PCR also showed that the level of lncRNA AC010761.9 expression was higher in the three GA cell lines (BGC-823, SGC-7901, and MGC-803) compared to normal gastric cells (GES-1 cells; *p* < 0.05) (Fig. 3). All of these findings indicated that lncRNA AC010761.9 is over-expressed in GA tissues and cell lines.

### Relationships between the level of lncRNA AC010761.9 expression and clinical pathologic factors

Among the 145 patients investigated in the present study, all were diagnosed with GA, and 129 patients had detailed clinical pathologic data. The clinical value of the expression of lncRNA AC010761.9 was then evaluated according to the level of expression and clinical pathologic factors. As depicted in Table 1, the elevated expression of this lncRNA was related with a tumor size ≥ 4 versus < 4 cm (*p* = 0.028) and degree of differentiation (*p* = 0.047), but not related with other clinical or pathologic factors, including age, gender, tumor location, lymphatic metastasis, and venous and perineural invasion (Table 1). Data from multivariate analysis further exhibited that lncRNA AC010761.9 expression was related with patient age (*p* = 0.043) and the degree of tumor differentiation (*p* = 0.015; Table 2). These results indicated that lncRNA AC010761.9 may be GA-associated lncRNA.

**Table 2:** Relationships between lncRNA AC010761.9 expression (ΔCt value) and patient clinical pathologic factors analyzed by univariate and multivariate

Characteristics	Univariate	Multivariate
	*p* value	*p* value
Diameter	0.028	0.255
Differentiation	0.047	0.015
Invasion	0.269	0.196
Lymphatic metastasis	0.597	0.777
Venous invasion	0.808	0.063
Perineural invasion	0.399	0.222
Location	0.621	0.473
Age	0.162	0.078
Sex	0.620	0.043

### Relationships between the level of lncRNA AC010761.9 expression and serum and IHC GA markers

As depicted in Table 1 and Additional file 1: Table S1, the relationships between the level of lncRNA AC010761.9 expression and serum CEA, CA19-9, and AFP concentrations and the ten IHC markers showed that lncRNA AC010761.9 expression was related with the serum carbohydrate antigen (CA19-9) and carcinoembryonic antigen (CEA) concentrations (*p* = 0.026 and *p* = 0.037, respectively), but not related to serum AFP concentrations and ten IHC markers (Table 1 and Additional file 1: Table S1).

### Relationships between the level of lncRNA AC010761.9 and TRAF4 mRNA expression in GA tissues and cell lines

**Fig. 4:**
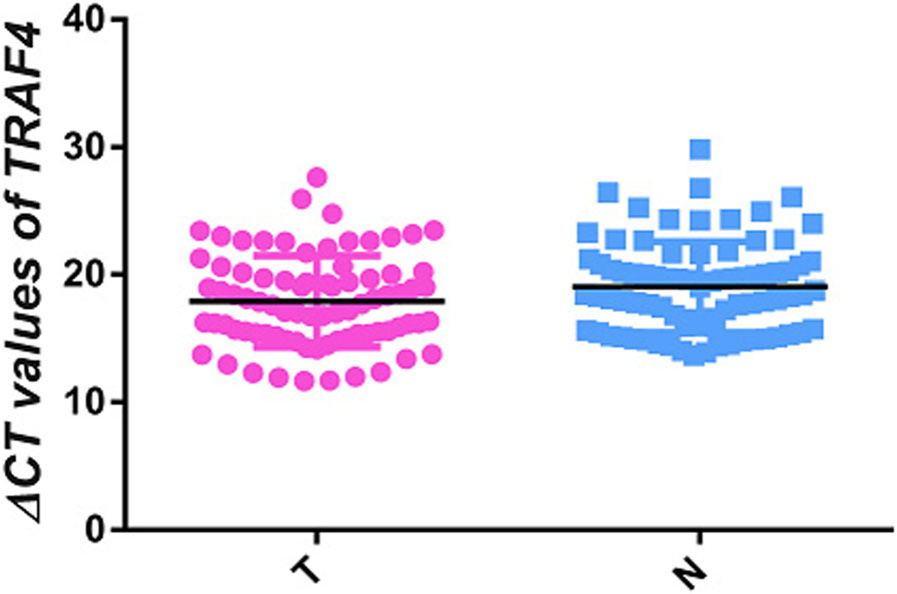
TRAF4 mRNA was over-expressed in GA tissues. *T* cancer tissues, *N* matched non-cancer tissues. T versus N, *p* < 0.05. The data were from 78 cases of GA. The higher the ΔCt values, the lower the TRAF4 mRNA expression. Data were obtained from three independent tests

**Fig. 5:**
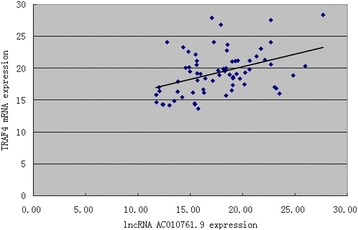
The expression of lncRNA AC010761.9 showed a positive correlation with the expression of TRAF4 mRNA. *r* = 0.385 and *p* < 0.01 were obtained by Pearson correlation analysis

TRAF4 has been observed to participate in several human cancer developments [11, 12]. The TRAF4 gene is speculated to be the potential sense strand gene of lncRNA AC010761.9, thus the level of its expression in 78 GA tissues and matched non-GA tissues was also investigated and related to the level of lncRNA AC010761.9 expression in the same tissues. As depicted in Fig. 4, the expression of TRAF4 mRNA in 54 of 78 GA tissues was higher than the matched non-GA tissues with a mean over-expressed rate of 69.2% (*p* < 0.05) and an elevated fold up to 10.04. The results from Pearson correlation analyses showed that the level of lncRNA AC010761.9 expression was to some degree a positive correlation to the expression of the potentially associated gene (TRAF4) mRNA in GA tissues (*r* = 0.385, *p* < 0.01)(Fig. 5). Additionally, the expression levels of lncRNA AC010761.9 and TRAF4 mRNA among the three GA cell lines (BGC-823, SGC-7901, and MGC-803) also exhibited similarly over-expressed trend compared with those in GES-1 cells.

## Discussion

Currently, a number of studies have shown that abnormal expression of lncRNAs is associated with cancer progression and prognosis, such as T staging, N staging, tumor size, invasion, and survival [14, 15]. Therefore, lncRNAs are regarded as a new biomarker and therapeutic target for various human cancers. Gastric cancer is a typical cancer, which frequently has dysregulated expression of lncRNAs. Abnormal expression of lncRNAs has been identified to play the roles of oncogenes or inhibiting genes exacerbating or attenuating gastric cancer development [16, 17]. In the current study, we also identified a lncRNA: LncRNA AC010761.9 may be GA-related.

LncRNA AC010761.9 is a RNA located on human chromosome 17 with a total length of 521 bp. LncRNA AC010761.9 does not have the ability of coding for protein, which is a new lncRNA and unreported in GA or other cancers. The level of this lncRNA expression was significantly up-regulated in GA tissues compared to normal tissues and was also significantly up-regulated in MGC803, BGC823, and SGC7901 cells. Over-expressed lncRNA AC010761.9 was related with tumor size and degree of GA differentiation, as well as related to serum CEA and CA199 levels of the digestive system tumor markers.

Tumor size and the degree of differentiation are associated with GA progression. Therefore, a lncRNA that is linked to these two pathologic factors may have important clinical value. CEA and CA19-9 are the two most widely confirmed and used serum digestive cancer markers worldwide [18]. If a lncRNA is associated with these two markers, the lncRNA may have the potential to be combined with CEA and CA19-9 levels and used for GA diagnosis or treatment monitoring.

Because the lncRNA itself does not encode the protein, but functions in the regulation of gene expression, we further investigated the expression level of the potentially associated gene (TRAF4 gene) of this lncRNA in GA tissues and cell lines and found that the level of TRAF4 mRNA was significantly up-regulated compared to normal tissues and was also higher expression in the three GA cell lines (MGC803, BGC823, and SGC7901) than normal gastric epithelial cells (GES-1). The level of lncRNA AC010761.9 expression in GA tissues and cell lines showed a positive correlation or similarly up-regulated trend with the potentially associated gene TRAF4 mRNA level. Therefore, lncRNA AC010761.9 could have the potential to play a biological role in GA by regulating the expression of TRAF4. Because the samples used in the current study were not sufficiently large and the relationship between the level of lncRNA AC010761.9 and TRAF4 mRNA expression value was lower, further investigations are needed to confirm this point, including the use of a larger tissue sample size and gene or siRNA transfection techniques to increase or decrease lncRNA AC010761.9 expression to observe the change in expression of TRAF4 mRNA.

TRAF4 is one of the members of the TRAF family of proteins [19]. It has been confirmed that TRAF4 is over-expressed in several different human cancers [19, 20]. TRAF4 is also known to play a major role in embryonic development, cell polarity, and apoptosis and regulation of reactive oxygen species (ROS) production [21]. TRAF4 and β-catenin exhibit a mutual relationship of interaction [22]. β-catenin was first recognized as an adhesion factor, and later studies showed that β-catenin is also a multifunctional protein [23], which is widely found in various types of cells, such as endothelial cells, fibroblasts, and osteoblasts and is involved in the regulation of cell proliferation, differentiation, and apoptosis. An abundance of data has shown that β-catenin is closely related with malignant tumor development and invasion and is also transfer-related [24]. β-catenin has been shown to have main functions on mediating cell adhesion and gene expression [25]. Additionally, β-catenin plays an important role in increased oncogene c-myc gene expression, leading to carcinogenesis [26]. TRAF4 is identified to function in enhancing β-catenin-related transcription and protecting the β-catenin protein to be degraded by the p53 gene-mediated degradation pathway. TRAF4 can also regulate the activation of NF-κB [27]. NF-kB activation can promote cell growth, thus many anti-tumor drugs target on NF-kB, which is regarded as the treated target of many different cancers [28]. TRAF4 can also regulate the formation of reactive oxygen species and is associated with JNK activation [29]. All these findings suggest that lncRNA AC010761.9 was identified as GA-related lncRNA, which may be associated with an increase in TRAF4 mRNA expression.

## Conclusion

In conclusion, the results from the present study indicated that lncRNA AC010761.9 is significantly up-regulated in GA tissues and GA cell lines. The expression of lncRNA AC010761.9 and TRAF4 in gastric carcinoma was positively correlated, which suggests that lncRNA AC010761.9 may play a biological role in the development and progression of GA possibly by regulating the expression of TRAF4. The expression level of lncRNA AC010761.9 was significantly correlated with the degree of tumor differentiation and tumor size and also correlated with serum CEA and CA199 levels of digestive system tumor markers, all of which indicate lncRNA AC010761.9 could be used as a new biomarker and a new target for the treatment of GA.

## Additional files

Additional file 1: Table S1. Relationship between lncRNA AC010761.9 expression (ΔCt value) and GA immunohistochemical markers (DOCX 15 kb)
