# Supplementary material for: Evaluation of the expression and clinical value of lncRNA AC010761.9 in human gastric adenocarcinoma
Source: World J Surg Oncol. 2018 Mar 2;16:40. doi: 10.1186/s12957-017-1289-y (PMC5833146; doi:10.1186/s12957-017-1289-y)
Supplement: Supplementary file 1 — Relationship between lncRNA AC010761.9 expression (ΔCt value) and GA immunohistochemical markers (DOCX 15 kb) [file 12957_2017_1289_MOESM1_ESM.docx]

**Relationship between lncRNA AC010761.9 expression（ΔCt value）and GA immunohistochemical markers**

Characteristcs No.of case(%) Mean±SD p value

VEGF

0 10(7.7) 19.43±3.647 0.953

1 13(10.1) 18.57±2.180

2 31(24.0) 18.80±3.278

3 54(41.9) 18.70±3.128

4 21(16.3) 18.47±3.250

C-erbB-2

0 106(82.2) 18.76±3.171 0.951

1 7(5.4) 18.41±1.310

2 5(3.9) 19.23±0.959

3 11(8.5) 18.38±4.036

TS

0 19(14.7) 18.83±2.662 0.678

1 47(36.4) 18.90±3.759

2 47(36.4) 18.81±2.807

3 16(12.4) 17.83±2.325

BRCA1

0 10(7.7) 18.61±1.777 0.837

1 28(21.7) 17.18±3.691

2 60(46.5) 18.98±3.143

3 29(22.5) 18.83±2.915

4 2(1.6) 17.88±2.178

ERCC1

0 22(17.0) 18.05±3.574 0.309

1 19(14.7) 19.08±4.485

2 35(27.3) 18.23±2.466

3 33(25.6) 18.81±2.696

4 20(15.5) 19.86±2.509

RRM1

0 73(56.6) 18.87±3.249 0.952

1 6(4.7) 18.92±4.100

2 15(11.6) 18.71±3.861

3 29(22.5) 18.49±2.240

4 6(4.6) 17.96±2.659

Ki67

1 15(11.6) 18.31±2.244 0.900

2 39(30.2) 18.97±3.812

3 42(32.6) 18.78±2.984

4 33(25.6) 18.57±2.751

Syn

0 107(82.9) 18.87±3.308 0.496

1 13(10.1) 17.49±1.812

2 4(3.1) 18.42±1.572

3 5(3.9) 19.14±1.203

CD56

0 111(86.0) 18.73±3.284 0.981

1 13(10.1) 18.51±1.947

2 2(1.6) 19.45±0.544

3 3(2.3) 18.93±1.065

CgA

0 125(96.9) 18.77±3.129 0.383

1 4(3.1) 17.39±2.178
